# Supplementary material for: Phase-shifting the circadian glucocorticoid profile induces disordered feeding behaviour by dysregulating hypothalamic neuropeptide gene expression
Source: Commun Biol. 2023 Sep 29;6:998. doi: 10.1038/s42003-023-05347-3 (PMC10541449; doi:10.1038/s42003-023-05347-3)
Supplement: Supplementary file 2 — Supplementary Information [file 42003_2023_5347_MOESM2_ESM.pdf]

S Fig. 1

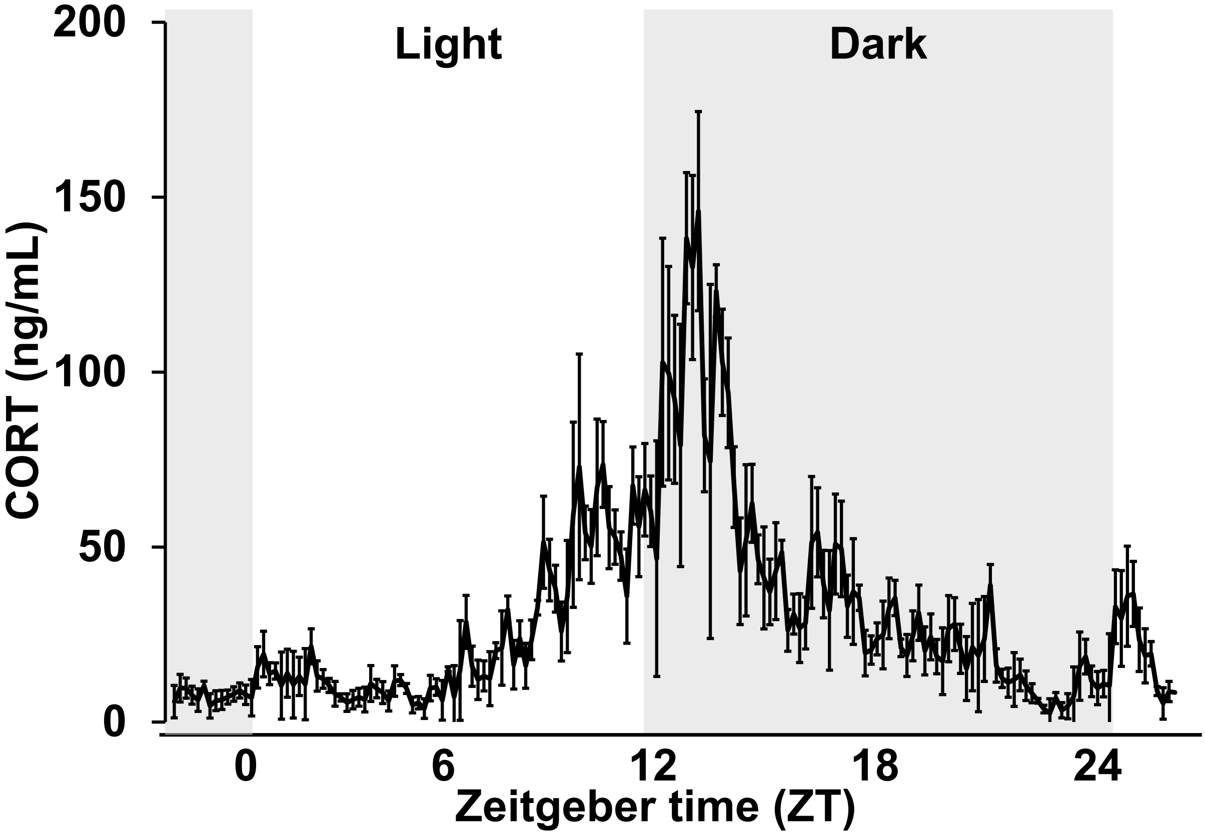

S Fig. 1 Circadian profile of plasma corticosterone (CORT) concentration.
Plasma CORT was measured by automated blood sampling system of adrenal-intact rats (n=14).

S Fig. 2

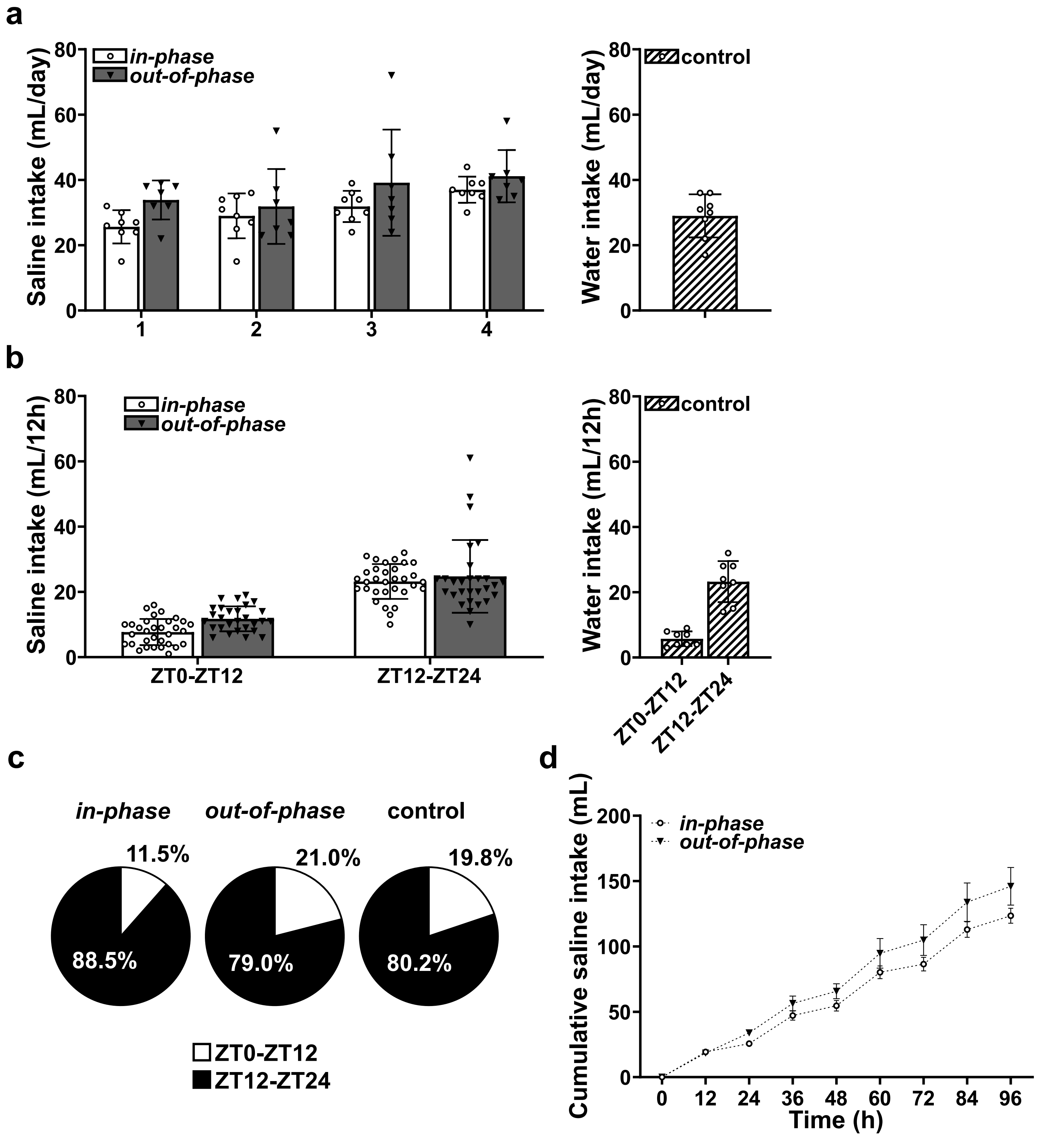

S Fig. 2 Timing of water intake is unaffected when corticosterone is *out-of-phase* with circadian cues.

(a) Total water intake (mL/day) over the course of the experiment. (b) Timing of water intake (mL/12h) is unaffected by corticosterone infusion pattern. (c) Percentage of water intake during light period (ZT0-ZT12) and dark period (ZT12-ZT24) for *in-phase*, *out-of-phase*, and control groups. (d) Cumulative measurements throughout the experiment. Data are presented as mean ± SD, with individual datapoints representing biological repeats shown on each graph (n=6-8).

S Fig. 3

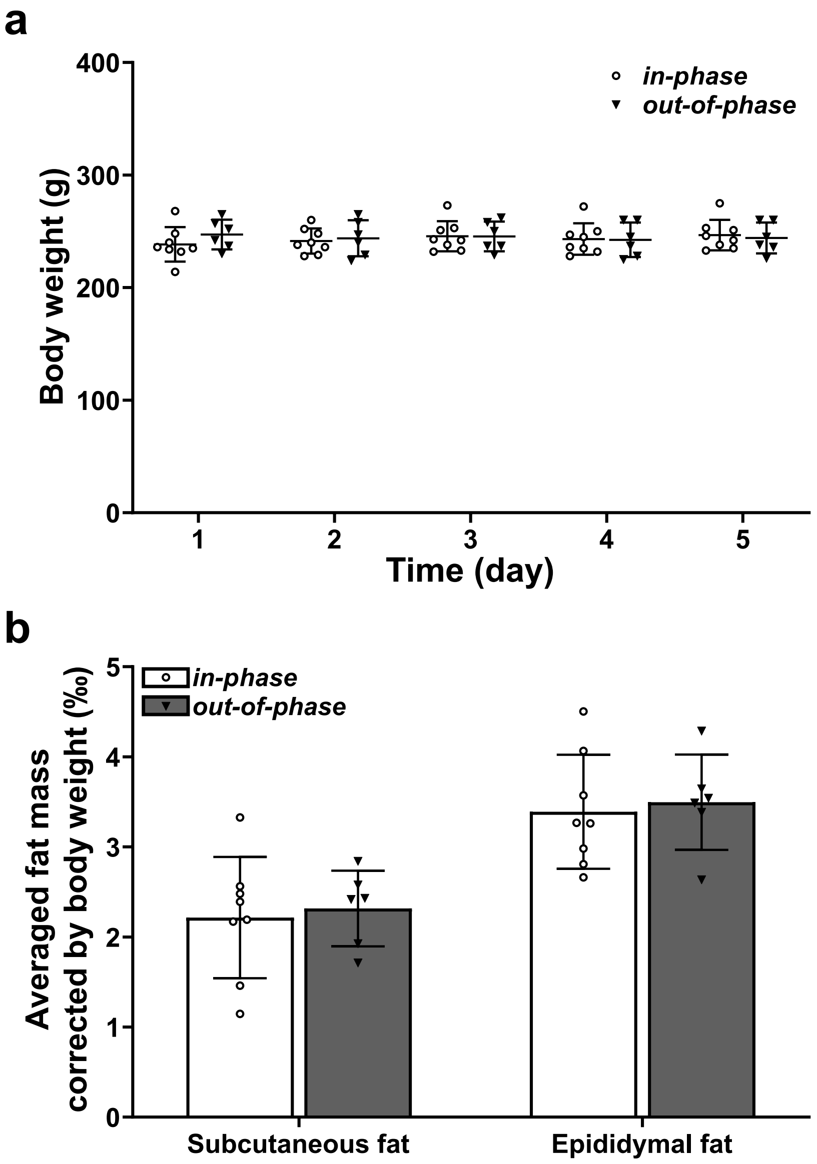

S Fig. 3 Body weight, subcutaneous fat mass, and epididymal fat mass are unaffected when corticosterone is *out-of-phase* with circadian cues.
(a) Body weight (g) over the course of the experiment. (b) Subcutaneous fat mass and epididymal fat mass were measured and compared (corrected by body weight) at the end of the experiment. Data are presented as mean ± SD, with individual datapoints representing biological repeats shown on each graph (n=6-8).

S Fig. 4

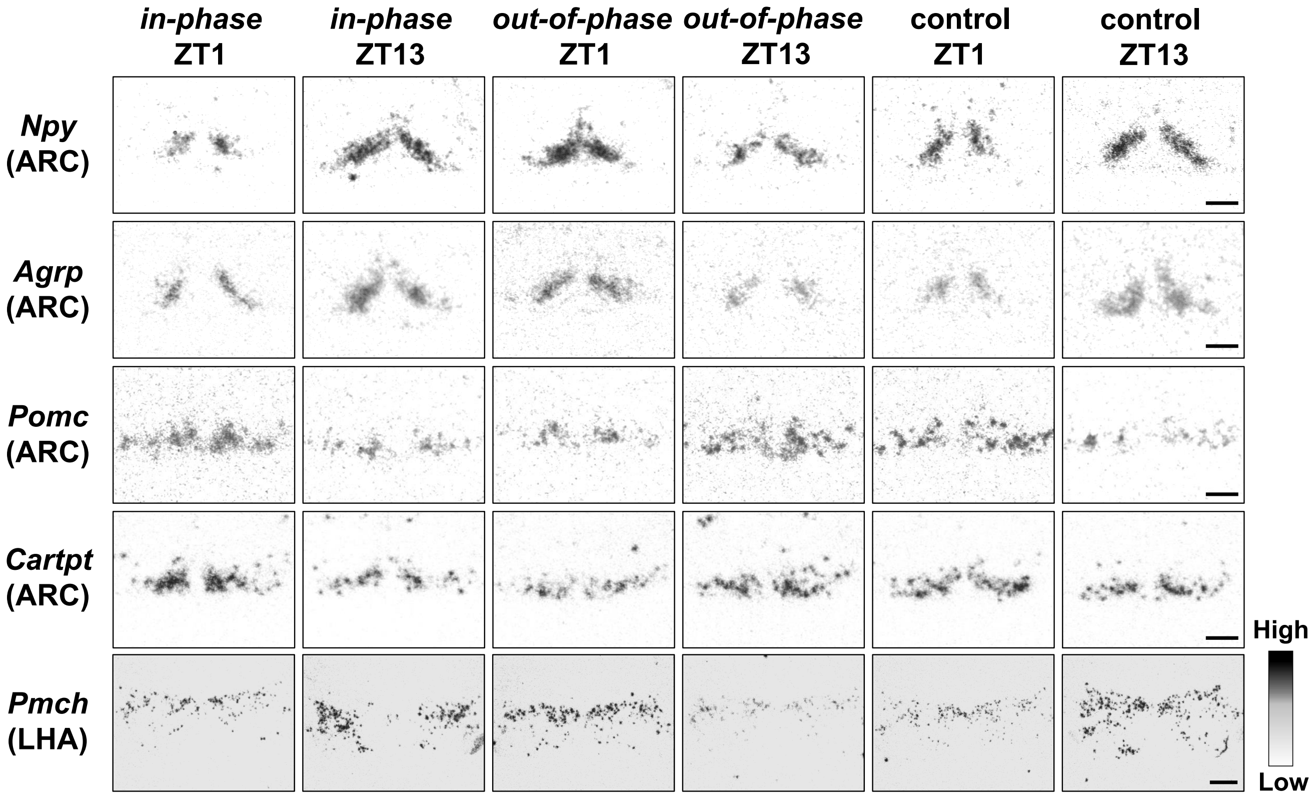

S Fig. 4 Representative images of *in situ* hybridization histochemistry (ISHH) in the arcuate nucleus (ARC) and lateral hypothalamic area (LHA).

ISHH images obtained from each experimental group. Scale bars, 200 um (in the ARC), and 1 mm (in the LHA). *Npy*, *neuropeptide Y*; *Agrp*, *agouti-related peptide*; *Pomc*, *proopiomelanocortin*; *Cartpt*, *cocaine and amphetamine regulated transcript prepropeptide*; *Pmch*, *pro-melanin concentrating hormone*.

S Fig. 5

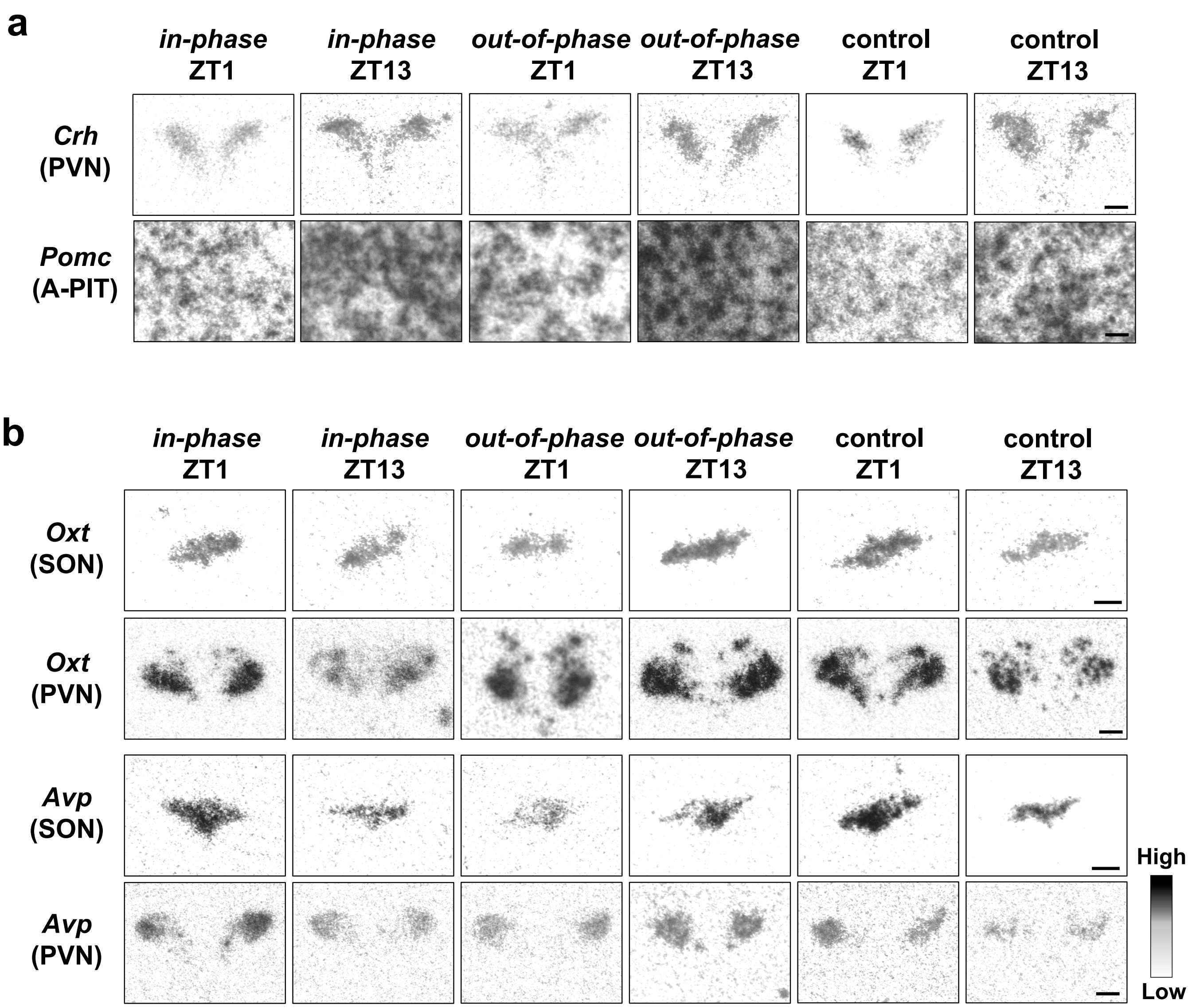

S Fig. 5 Representative images of *in situ* hybridization histochemistry (ISHH) in the supraoptic nucleus (SON), paraventricular nucleus (PVN), and anterior pituitary (A-PIT).

ISHH images obtained from each experimental group in the PVN and A-PIT (a) and SON and PVN (b). Scale bars, 200 um. *Crh*, *corticotrophin releasing hormone*; *Pomc*, *proopiomelanocortin*; *Oxt*, *oxytocin*; *Avp*, *arginine vasopressin*.

S Fig. 6

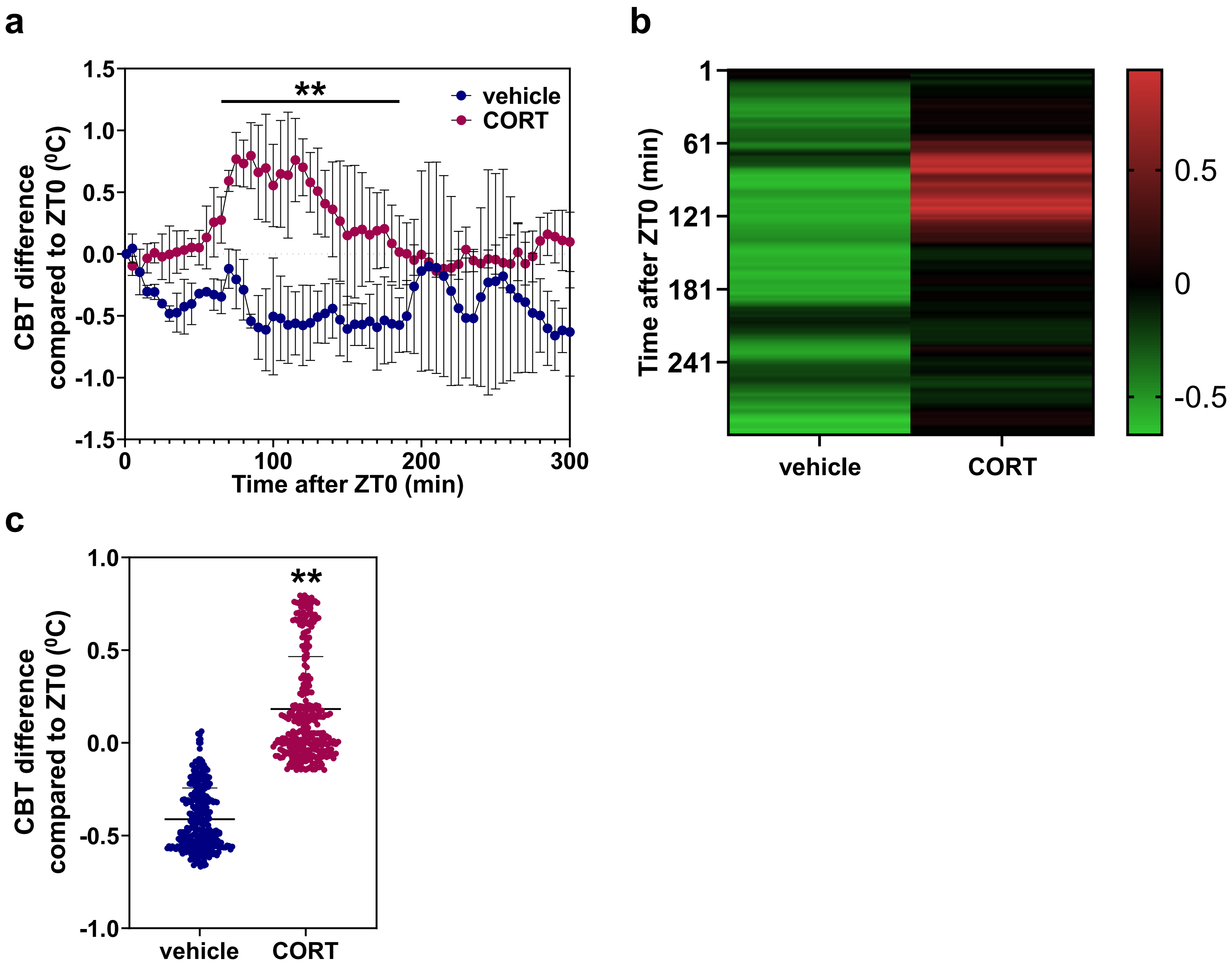

S Fig. 6 Core body temperature (CBT) is significantly increased following subcutaneous administration of corticosterone (CORT).
To eliminate the impact of feeding-induced CBT elevation, rats were deprived of food from ZT23 onwards. Subsequently, at ZT1, rats received subcutaneous injections of either vehicle or CORT (3mg/kg) (vehicle, n=2; CORT n=3), and their CBT was recorded using a telemetry probe until ZT5. (a) Changes in CBT compared to ZT0. Data are presented as mean +/- SD. (b) Heat map representation of CBT changes from ZT0 to ZT5. (c) Individual plots of CBT changes from ZT0 to ZT5. ***P*<0.01 vs. vehicle.

S Fig. 7

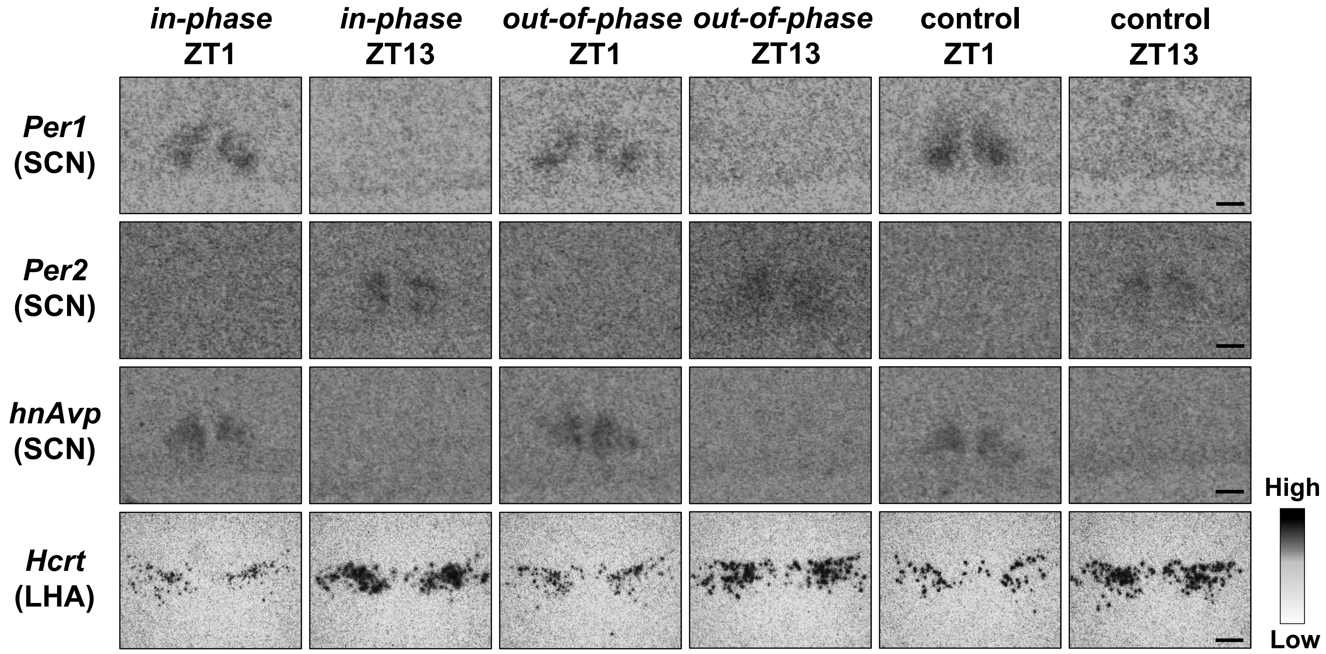

S Fig. 7 Representative images of *in situ* hybridization histochemistry (ISHH) in the suprachiasmatic nucleus (SCN) and lateral hypothalamic area (LHA). ISHH images obtained from each experimental group in the SCN and LHA. Scale bars, 200 um (in the SCN), 1 mm (in the LHA). *Per1*, *period1*; *Per2*, *period2*; *hnAVP*, *heteronuclear arginine vasopressin*; *Hcrt*, *hypocretin neuropeptide precursor*.
