# Supplementary material for: Phase-shifting the circadian glucocorticoid profile induces disordered feeding behaviour by dysregulating hypothalamic neuropeptide gene expression
Source: Commun Biol. 2023 Sep 29;6:998. doi: 10.1038/s42003-023-05347-3 (PMC10541449; doi:10.1038/s42003-023-05347-3)
Supplement: Supplementary file 5 — Reporting Summary [file 42003_2023_5347_MOESM5_ESM.pdf]

nature portfolio

Corresponding author(s): Becky Conway-Campbell

Last updated by author(s): Aug 18, 2023

Reporting Summary

Nature Portfolio wishes to improve the reproducibility of the work that we publish. This form provides structure for consistency and transparency in reporting. For further information on Nature Portfolio policies, see our Editorial Policies and the Editorial Policy Checklist.

Statistics

For all statistical analyses, confirm that the following items are present in the figure legend, table legend, main text, or Methods section.

n/a	Confirmed	
☐	☒	The exact sample size (n) for each experimental group/condition, given as a discrete number and unit of measurement
☐	☒	A statement on whether measurements were taken from distinct samples or whether the same sample was measured repeatedly
☐	☒	The statistical test(s) used AND whether they are one- or two-sided<br>*Only common tests should be described solely by name; describe more complex techniques in the Methods section.*
☒	☐	A description of all covariates tested
☐	☒	A description of any assumptions or corrections, such as tests of normality and adjustment for multiple comparisons
☐	☒	A full description of the statistical parameters including central tendency (e.g. means) or other basic estimates (e.g. regression coefficient) AND variation (e.g. standard deviation) or associated estimates of uncertainty (e.g. confidence intervals)
☐	☒	For null hypothesis testing, the test statistic (e.g. F, t, r) with confidence intervals, effect sizes, degrees of freedom and P value noted<br>*Give P values as exact values whenever suitable.*
☒	☐	For Bayesian analysis, information on the choice of priors and Markov chain Monte Carlo settings
☒	☐	For hierarchical and complex designs, identification of the appropriate level for tests and full reporting of outcomes
☒	☐	Estimates of effect sizes (e.g. Cohen's d, Pearson's r), indicating how they were calculated

Our web collection on statistics for biologists contains articles on many of the points above.

Software and code

Policy information about availability of computer code

Data collection	Not applicable.
Data analysis	Graph pad prism 9 was used for data analysis. All data were analyzed by student t test, one-way ANOVA, or two-way ANOVA followed by a post-hoc test for multiple comparisons. Statistical significance was set at P<0.05.

For manuscripts utilizing custom algorithms or software that are central to the research but not yet described in published literature, software must be made available to editors and reviewers. We strongly encourage code deposition in a community repository (e.g. GitHub). See the Nature Portfolio guidelines for submitting code & software for further information.

Data

Policy information about availability of data

All manuscripts must include a data availability statement. This statement should provide the following information, where applicable:

- Accession codes, unique identifiers, or web links for publicly available datasets
- A description of any restrictions on data availability
- For clinical datasets or third party data, please ensure that the statement adheres to our policy

The experimental data and materials are available from the corresponding authors, upon reasonable request.

Research involving human participants, their data, or biological material

Policy information about studies with human participants or human data. See also policy information about sex, gender (identity/presentation), and sexual orientation and race, ethnicity and racism.

Reporting on sex and gender	Not applicable.
Reporting on race, ethnicity, or other socially relevant groupings	Not applicable.
Population characteristics	Not applicable.
Recruitment	Not applicable.
Ethics oversight	Not applicable.

Note that full information on the approval of the study protocol must also be provided in the manuscript.

Field-specific reporting

Please select the one below that is the best fit for your research. If you are not sure, read the appropriate sections before making your selection.

☒ Life sciences ☐ Behavioural & social sciences ☐ Ecological, evolutionary & environmental sciences

For a reference copy of the document with all sections, see nature.com/documents/nr-reporting-summary-flat.pdf

Life sciences study design

All studies must disclose on these points even when the disclosure is negative.

Sample size	The number of animals used in this study followed the standard in similar studies in rodents. We used a minimum number of animals to judge consistency across subjects.
Data exclusions	No data were excluded from the analysis.
Replication	All results we obtained were consistent across the animals.
Randomization	Rats were randomly assigned in all experiments.
Blinding	The results were double-blinded in all experiments. All data were analysed by at least three different researchers.

Reporting for specific materials, systems and methods

We require information from authors about some types of materials, experimental systems and methods used in many studies. Here, indicate whether each material, system or method listed is relevant to your study. If you are not sure if a list item applies to your research, read the appropriate section before selecting a response.

Materials & experimental systems

n/a	Involved in the study	
☐	☒	Antibodies
☒	☐	Eukaryotic cell lines
☒	☐	Palaeontology and archaeology
☐	☒	Animals and other organisms
☒	☐	Clinical data
☒	☐	Dual use research of concern
☒	☐	Plants

Methods

n/a	Involved in the study	
☒	☐	ChIP-seq
☒	☐	Flow cytometry
☒	☐	MRI-based neuroimaging

Antibodies

Antibodies used	anti-GR antibody [2μg of 24050-1-AP (Proteintech)], anti-pSer5 RNA Pol2 [10µl; 39233 (ACTIVE MOTIF)]

Validation	All primary antibodies used in the present study have been validated in rats according to the manufacture's instruction and previous studies.

Animals and other research organisms

Policy information about [studies involving animals](); [ARRIVE guidelines]() recommended for reporting animal research, and [Sex and Gender in Research]()

Laboratory animals	Adult male Sprague-Dawley (SD) rats (220-240g, Harlan-Olac, Oxon, UK) were used.
Wild animals	No wild animals were used in this study.
Reporting on sex	Certainly, conducting experiments using female rats in the future will be both interesting and necessary in a translational sense. However, this study was performed using only male rats. The reason for this was because -to perform the current experiments in a way that is meaningful- we would need to control for estrous cycle stage, which would require a large n number of animals for each experiment. This was simply not possible at the time. We are hopeful that interest in our first study in males will enable further funding, which we can use to assess how circadian misalignment affects females. In particular, it will be really interesting to assess different life stages especially older females as hormones levels change.
Field-collected samples	No field collected samples were used in this study.
Ethics oversight	All animal procedures conformed to the regulations of, and were approved by, the Home Office, UK and the Institution (University of Bristol, UK).

Note that full information on the approval of the study protocol must also be provided in the manuscript.
